# 80 Hz auditory steady state responses (ASSR) elicited by silent gaps embedded within a broadband noise

**DOI:** 10.3389/fneur.2023.1221443

**Published:** 2023-07-13

**Authors:** Seiichi Kadowaki, Takashi Morimoto, Marta Pijanowska, Shuji Mori, Hidehiko Okamoto

**Affiliations:** ^1^Department of Physiology, International University of Health and Welfare Graduate School of Medicine, Narita, Japan; ^2^Department of Audiological Engineering, RION Co., Ltd., Tokyo, Japan; ^3^Office of Medical Education, International University of Health and Welfare School of Medicine, Narita, Japan; ^4^Graduate School of Humanities and Sociology, University of Tokyo, Tokyo, Japan; ^5^Department of Informatics, Graduate School of Information Science and Electrical Engineering, Kyusyu University, Fukuoka, Japan

**Keywords:** auditory steady state response (ASSR), electroencephalography (EEG), gap, human, temporal processing, speech

## Abstract

**Introduction:**

Although auditory temporal processing plays an important role in speech comprehension, it cannot be measured by pure tone audiometry. Auditory temporal resolution is often assessed by behavioral gaps-in-noise test. To evaluate whether auditory temporal resolution could be objectively assessed, we measured the auditory steady state response (ASSR) elicited by silent gaps embedded within broadband noises at 80 Hz.

**Methods:**

We prepared six sound types as test stimuli. One was a continuous broadband noise without a silent interval as a control stimulus and the others were broadband noises with 80 Hz silent intervals of 0.4, 0.8, 1.6, 3.1, and 6.3 ms.

**Results:**

Significant ASSRs were recorded only when the gap length was longer than the behavioral thresholds and the ASSR amplitude increased as the gap length increased.

**Conclusion:**

Eighty Hertz gap-evoked ASSR appears to reflect the neural activity related to the auditory gap processing and may be used as an objective measure of auditory temporal resolution in humans.

## 1. Introduction

Time is a very important variable in hearing because all sounds vary in frequency and amplitude over time. Complex natural sound signals such as speech and music can be decomposed into slowly varying “envelope” and rapidly oscillating “fine structure.” Out of the two, envelope plays a more important role in speech comprehension (1). Auditory temporal resolution is the ability to detect temporal changes of sound stimuli and to correctly recognize the envelope of sound signals. The temporal resolution of the auditory system is often assessed by the gap detection test. This test uses sound stimuli with silent gaps to estimate the minimum perceivable gap length (2, 3). It should be noted that this method requires the participant to take an action in response to the sound signal whenever they perceived the gap. As such, this method is not objective since the participant's personality, varying levels of concentration and dexterity necessary for the physical action of pressing the button may affect the results to some extent. Therefore, many studies currently aim to find objective measures of the temporal resolution using neuroimaging techniques.

Previous studies have shown that gaps embedded in continuous tones elicit various types of event related potentials. In an experiment by Uther et al. (4), continuous 2,000 Hz pure tones inserted with silent gaps of 3, 5, and 7 ms evoked mismatch negativity (MMN) and the MMN amplitude was proportional to the gap length. Electrophysiological studies have shown that the main sources of MMN are located close to the primary and secondary auditory cortex (5). Furthermore, Werner et al. (6) found that the behavioral gap detection threshold is similar to the neurophysiological gap detection threshold which was estimated by the sensitivity of the V wave of the auditory brainstem response elicited by silent gaps embedded within a broadband noise. The neural generator of V wave is thought to be the inferior colliculus (7). It remains elusive whether it is the cortex or the brainstem that plays a more important role in auditory gap detection.

In our previous study (8), we have obtained clear 40 Hz auditory steady state responses (ASSRs) elicited by the silent gaps of 3.125, 6.25, and 12.5 ms embedded within a broadband noise. The ASSR is one of the objective hearing tests used in clinical practice. Its neural sources and sensitivity depend on the modulation frequency. The 40 Hz ASSR appears to originate in the region spanning from the primary auditory cortex (9). It is considered to have a higher signal-to-noise ratio while the subject is awake (10). On the other hand, the 80 Hz ASSR appears to originate primarily from subcortical sources (11), and sleep has little effect on the response (12).

In the previous study, we demonstrated that gaps embedded within a broadband noise elicited clear 40 Hz ASSR; however, there has been no report that investigated the ASSR elicited by gap stimulus presented at the 80 Hz rate and compared the gap-evoked ASSR and the behavioral gap detection thresholds. The 80 Hz ASSR has the advantage of being an objective measure used in clinical practice because it can be measured reliably even during sleep (13). In this study, we conducted an experiment to verify whether the ASSR can be elicited by 80 Hz silent gaps of different lengths that are below and above the behavioral threshold. The results would contribute to developing a non-invasive objective measure of auditory temporal resolution in humans.

## 2. Methods

### 2.1. Participants

Twenty-one students (10 males) were recruited at the International University of Health and Welfare for this experiment. Their ages ranged from 18 to 31 (median 20). Eighteen participants were right-handed and three were left-handed, and all had normal hearing and no neurological or psychiatric disorders. They were fully informed about the study and gave written informed consent for their participation. The present study was approved by the Ethics Committee of the International University of Health and Welfare, School of Medicine and conformed to The Code of the World Medical Association (Declaration of Helsinki).

### 2.2. Stimuli and experimental design for electroencephalography recording

**Figure 1:**
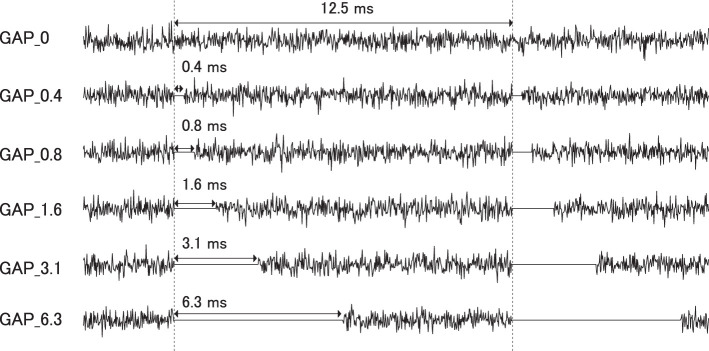
Stimulus conditions. Exemplary sound waveforms of test sound stimuli with silent intervals of 0 ms (GAP_0), 0.396 ms (GAP_0.4), 0.792 ms (GAP_0.8), 1.563 ms (GAP_1.6), 3.125 ms (GAP_3.1), and 6.25 ms (GAP_6.3) are depicted from top to bottom.

**Figure 2:**
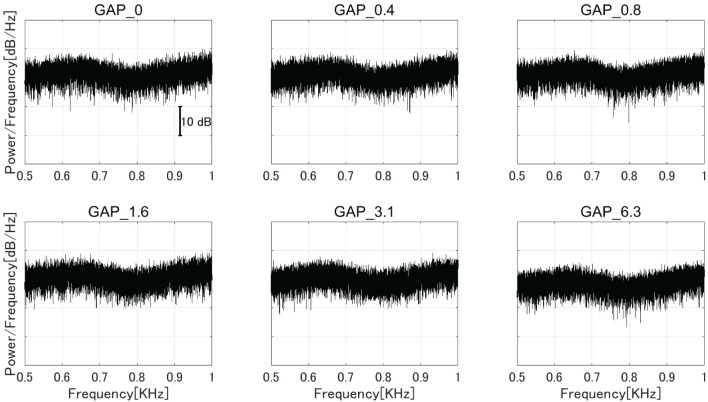
The amplitude spectra of the sound stimuli measured at the earpiece by using an ear simulator [GAP_0 **(top left)**, GAP_0.4 **(top center)**, GAP_0.8 **(top right)**, GAP_1.6 **(bottom left)**, GAP_3.1 **(bottom center)**, and GAP_6.3 **(bottom right)**].

Six types of white noise of 1 min duration were used as sound stimuli (sampling rate: 48,000 Hz). One type was a white noise without a silent interval as a control stimulus (GAP_0), and the others were white noises with 80 Hz silent intervals of 0.396 (GAP_0.4), 0.792 (GAP_0.8), 1.563 (GAP_1.6), 3.125 (GAP_3.1), and 6.25 ms (GAP_6.3; Figure 1 and Supplementary Audios 1–6). The stimuli were randomly played for 30 min, resulting in 5 min of each GAP condition (1 min × 5 times × 6 noise types). All auditory stimuli were designed in the MATLAB environment (The MathWorks Inc., MA, USA). Participants were presented with stimuli at an intensity of 70 dBA SPL via ER-3A insert earphones (Etymotic Research Inc., IL, USA). Figure 2 displays the amplitude spectra of the sound stimuli measured at the earpiece by using Ear Simulator TYPE 4157 (Bruel & Kjaer Sound & Vibration Measurement A/S, Denmark). EEG recordings were performed with participants seated comfortably in a silent electromagnetically shielded room. They were instructed to watch a silent movie with captions during experiments.

### 2.3. Behavioral data

The detection threshold of 80 Hz gap inserted sound stimuli and single gap inserted stimuli were measured in all participants. An adaptive, three-alternative forced-choice, two-down, one-up procedure was used to track the 70.7% correct rate for gap detection threshold (GDT) determination as described in detail in the previous studies (14, 15). The threshold for the length of detectable gap was tested at an intensity of 70 dBA SPL via ER-3A insert earphones, same as in the ASSR measurements. The duration of the white noise (sampling rate: 48,000 Hz) was set to 500 ms. In the detection threshold condition for a single gap inserted stimulus, each test sound contains only one silent gap in the middle, whereas in the detection threshold condition for an 80 Hz gap inserted sound stimulus, each test sound contains 40 silent gaps because the gaps are embedded at 80 Hz. The inter-stimulus interval between two successive test sounds was 500 ms. The gap length started from 7 ms. The step size was set to 1 ms in the first four reversals and 0.5 ms thereafter. The measurements were continued for 12 reversals, and the threshold was estimated as the mean of the values for the last eight reversals. Thresholds were measured twice, and the mean of the two measurements was used as the detection threshold.

### 2.4. Data acquisition and analysis

Sound stimuli were presented via Multi Trigger System Ver.2 (MTS0410, Medical Try System, Co., Ltd., Japan), which simultaneously sent triggers to Neurofax EEG1200 (Nihon Koden, Co., Ltd., Japan). We used six types of triggers (GAP_0, GAP_0.4, GAP_0.8, GAP_1.6, GAP_3.1, and GAP_6.3) and, apart from the control condition (GAP_0), the triggers were synchronized with the gap offset (or the onset of the noise segment). The EEG signals were recorded using a Neurofax EEG1200 system at a sampling rate of 1,000 Hz. The recording electrodes (Ag/AgCl) were located at Cz according to the international 10–20 system. The averaged signal of two electrodes placed on both mastoids was used as a reference electrode and the ground electrode was located around the forehead midpoint. Electrode impedance was maintained below 15 kΩ. Recorded EEG data were exported as ASCII files and were analyzed offline using Matlab R2020a and EEGLAB (16).

For EEG waveforms, a fast Fourier transform (FFT) was computed in each condition and amplitude spectra were extracted after removing the power line fluctuations at 50 Hz using the Clean-Line plugin for EEGLAB. In order to obtain ASSR, an epoching procedure was applied to the EEG signals. While the 80 Hz cycle necessitates the triggers to be placed at 12.5 ms intervals, this was difficult due to 1,000 Hz sampling rate. This problem was resolved by placing a marker every 25 ms in a 40 Hz cycle and calculating two cycles as one epoch. One minute sound stimulus contained 2,400 epochs, and for each GAP condition, the sound stimulus was presented 5 times, resulting in 12,000 epochs. A total of 60,000 epochs were labeled in all GAP conditions of each participant. EEG waveforms were bandpass filtered (70–90 Hz) offline and epochs of 0–24 ms (25 sampling points) from the markers were separately averaged after artifact rejection (set to a threshold of ± 20 μV) for each GAP condition (GAP_0, GAP_0.4, GAP_0.8, GAP_1.6, GAP_3.1, and GAP_6.3) for each participant. The obtained ASSR waveforms were fitted into the 80 Hz sinusoidal curves and the amplitudes were used for the statistical analysis.

Next, we calculated component synchrony measure (CSM) (17) and estimated GDT from the CSM obtained at Cz. The acquired EEG was filtered by a bandpass filter with cutoff frequencies of 79 and 81 Hz. Then the filtered EEG was divided into 600 epochs of 500 ms length and grouped into 10 groups, each containing 60 epochs. We obtained ten averaged waveforms based on those 60 epochs and the CSM is calculated using the following formula:


$$\begin{array}{l} CSM(m)=(\frac{1}{10}\sum_{k=1}^{10}\sin\psi_{k}[m]{)}^{2}+(\frac{1}{10}\sum_{k=1}^{10}\cos\psi_{k}[m]{)}^{2} \end{array}$$

Where, ψ denotes phase of ten averaged waveforms (*k* = 1, 2, 3, …10) and *m* denotes frequency. CSM value varies from 0 to 1. The value is equal to 1 if the phases of epochs are the same and approaches 0 when the phases change randomly. The criterion for the presence of response is set at *M*+3*SD* (= 0.385), where *M* denotes the mean of CSM value for non-response and *SD* denotes standard deviation. The obtained CSM functions as silent interval lengths were approximated by a sigmoid function, and the silent interval length whose obtained sigmoid function exceeded 0.385 was defined as GDT estimated from the CSM values.

### 2.5. Statistical analysis

In order to minimize the inter-individual differences, the ASSR amplitudes were normalized with respect to the mean ASSR amplitude averaged across all GAP conditions (GAP_0, GAP_0.4, GAP_0.8, GAP_1.6, GAP_3.1, and GAP_6.3) for each participant. Thereafter, the normalized ASSR amplitudes were evaluated by means of a one-way analysis of variance (ANOVA) using gap length (Gap_0, GAP_0.4, GAP_0.8, GAP_1.6, GAP_3.1, and GAP_6.3) as a factor and multiple-comparisons were analyzed using the Tukey's honestly significant difference test.

The CSMs calculated from EEG were similarly evaluated by means of a one-way ANOVA using gap length (Gap_0, GAP_0.4, GAP_0.8, GAP_1.6, GAP_3.1, and GAP_6.3) as a factor and multiple-comparisons were analyzed using the Tukey's honestly significant difference test.

The relationships between GDT estimated from the CSM values, single gap behavioral GDT, and 80 Hz gap behavioral GDT were evaluated based on correlation analysis.

## 3. Results

**Figure 3:**
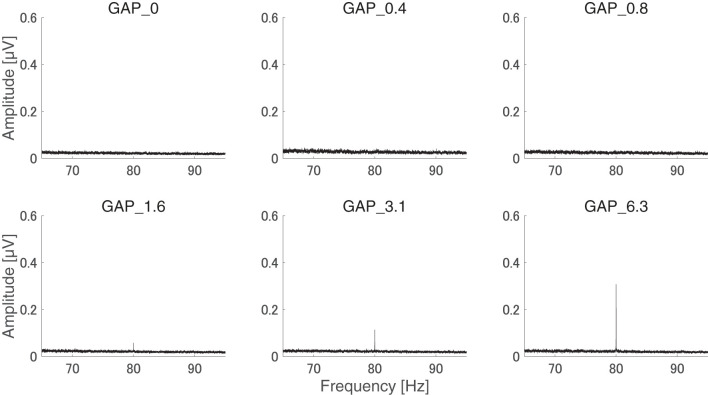
Group means of the electroencephalography (EEG) amplitude spectra. Grand averaged (*N* = 21) EEG amplitude spectra corresponding to GAP_0 **(top left)**, GAP_0.4 **(top center)**, GAP_0.8 **(top right)**, GAP_1.6 **(bottom left)**, GAP_3.1 **(bottom center)**, and GAP_6.3 **(bottom right)** are displayed. Clear induced brain responses are visible at 80 Hz in the GAP_1.6, GAP_3.1, and GAP_6.3 conditions.

**Figure 4:**
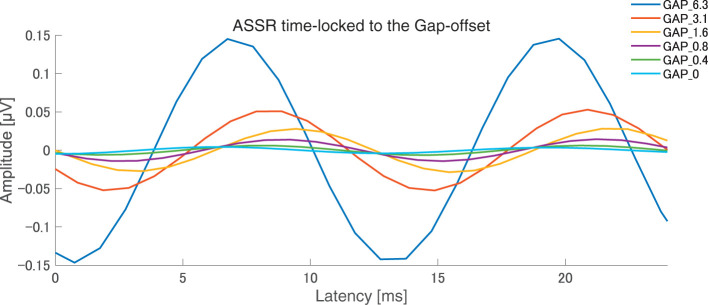
Grand averaged auditory steady state responses (ASSRs) elicited by 80 Hz silent gaps. The graph displays the grand-averaged waveforms of participants (*N* = 21). Blue, red, yellow, purple, green, light blue color lines represent GAP_6.3, GAP_3.1, GAP_1.6, GAP_0.8, GAP_0.4, and GAP_0 conditions, respectively (see legends in the right upper corner).

After the measurements were obtained from all participants, the collected data were analyzed. The average of the behavioral data of single gap GDT was 2.76 and the 95% confidence interval obtained by boot-strap resampling tests (iteration = 100,000) was 2.60–2.92. The average of the behavioral 80 Hz gap GDT was 0.994 and the 95% confidence interval obtained by boot-strap resampling tests (iteration = 100,000) was 0.902–1.101. For the EEG analysis, the mean rejection rate of the obtained epochs was 6.6%. Figure 3 shows grand averaged FFT waveforms under each condition. No clear response at 80 Hz was observed under GAP_0, GAP_0.4, and GAP_0.8 conditions in contrast to GAP_1.6, GAP_3.1, and GAP_6.3 conditions, where prominent responses at 80 Hz were observed. Figure 4 shows grand averaged EEG waveforms under each condition. Similar to the FFT results, no significant ASSR was evoked in the GAP_0, GAP_0.4, and GAP_0.8 conditions. In the GAP_1.6, GAP_3.1, and GAP_6.3 conditions, clear EEG was evoked and the amplitude increased as the gap length increased.

**Figure 5:**
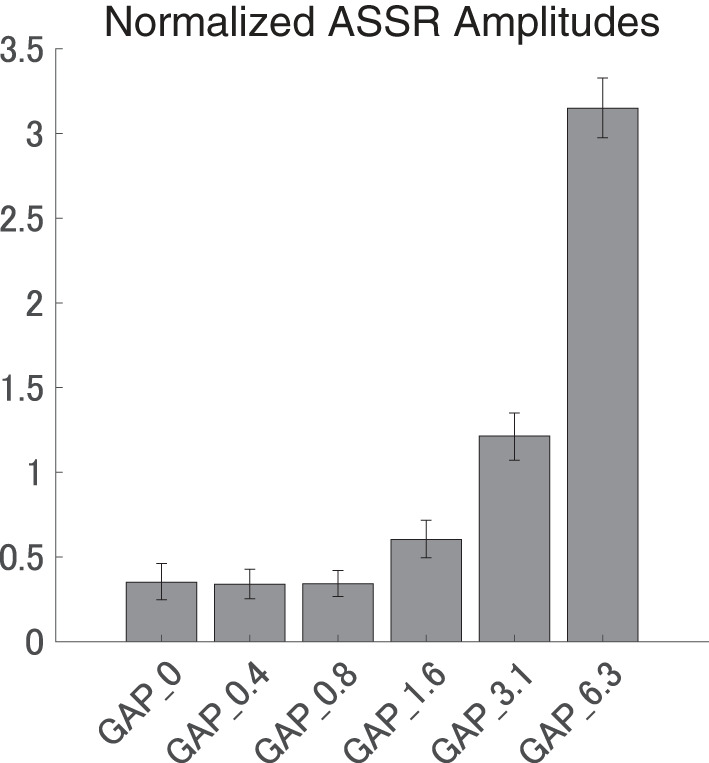
Group means of the normalized auditory steady state response (ASSR) amplitudes. Group means (*N* = 21) of the normalized ASSR amplitudes elicited by silent gaps of 0 ms (GAP_0), 0.396 ms (GAP_0.4), 0.792 ms (GAP_0.8), 1.563 ms (GAP_1.6), 3.125 ms (GAP_3.1), and 6.25 ms (GAP_6.3) embedded within broadband noises. The error bars denote 95% confidence intervals.

Figure 5 shows the mean normalized ASSR amplitude in each GAP condition together with the corresponding 95% confidence intervals obtained by boot-strap resampling tests (iteration = 100 000). A one-way ANOVA applied to the normalized ASSR amplitude revealed a significant main effect for gap length [*F*_(5, 120)_ = 299.66, *p* < 0.0001]. As shown in Table 1, the *post-hoc* multi-comparison revealed significant differences between GAP_0 and GAP_3.1 (*p* < 0.0001), GAP_0 and GAP_6.25 (*p* < 0.0001), GAP_0.4 and GAP_1.6 (*p* = 0.048), GAP_0.4 and GAP_3.1 (*p* < 0.0001), GAP_0.4 and GAP_6.25 (*p* < 0.0001), GAP_0.8 and GAP_3.1 (*p* < 0.0001), GAP_0.8 and GAP_6.25 (*p* < 0.0001), GAP_1.6 and GAP_3.1 (*p* < 0.0001), GAP_1.6 and GAP_6.25 (*p* < 0.0001), and GAP_3.1 and GAP_6.25 (*p* < 0.0001). The ASSR amplitudes gradually increased with an increase in the gap duration.

**Table 1:** *p*-values of *post-hoc* multi comparisons between GAP conditions using Tukey's multiple comparison test.

	**GAP length**	**0**	**0.4**	**0.8**	**1.6**	**3.1**
Normalized amplitude	0.4	1	–	–	–	–
	0.8	1	1	–	–	–
	1.6	0.069	0.048	0.054	–	–
	3.1	<0.0001	<0.0001	<0.0001	<0.0001	–
	6.3	<0.0001	<0.0001	<0.0001	<0.0001	<0.0001
CSM	0.4	0.840	–	–	–	–
	0.8	0.807	1	–	–	–
	1.6	0.011	0.0232	0.262	–	–
	3.1	<0.0001	<0.0001	<0.0001	<0.0001	–
	6.3	<0.0001	<0.0001	<0.0001	<0.0001	<0.0001

**Figure 6:**
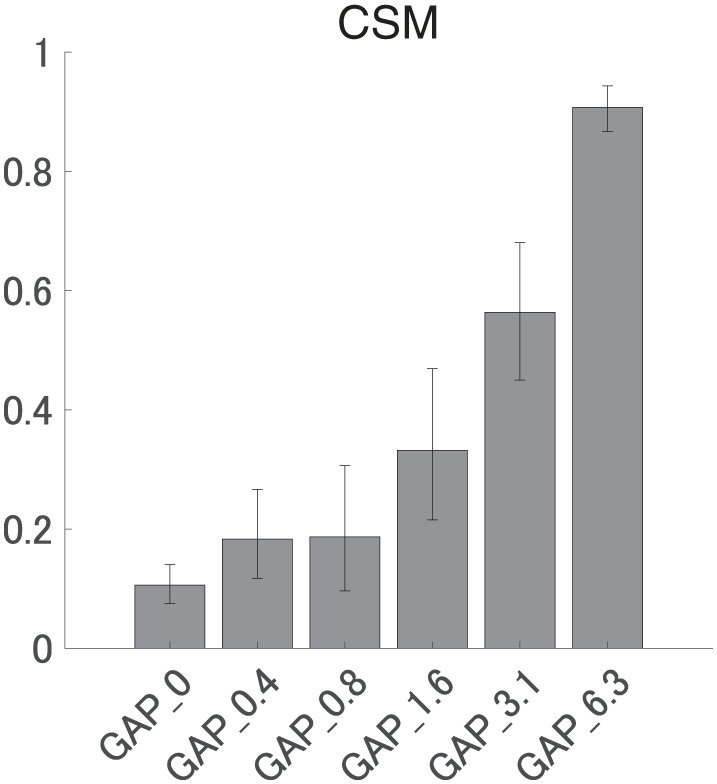
Group means of the component synchrony measure (CSM) of auditory steady-state response (ASSR). Group means (*N* = 21) of the CSM elicited by silent gaps of 0 ms (GAP_0), 0.396 ms (GAP_0.4), 0.792 ms (GAP_0.8), 1.563 ms (GAP_1.6), 3.125 ms (GAP_3.1), and 6.25 ms (GAP_6.3) embedded within broadband noises. The error bars denote 95% confidence intervals.

Figure 6 shows the mean CSMs calculated from EEG in each GAP condition together with the corresponding 95% confidence intervals obtained by boot-strap resampling tests (iteration = 100 000). For the results in GAP_1.6, GAP_3.1, and GAP_6.25, the mean CSM gradually increased as GAP length increased. A one-way ANOVA applied to the CSM revealed a significant main effect for gap length [*F*_(5, 120)_ = 41.33, *p* < 0.0001]. As shown in Table 1, the *post-hoc* multiple comparison revealed significant differences between GAP_0 and GAP_1.6 (*p* = 0.011), GAP_0 and GAP_3.1 (*p* < 0.0001), GAP_0 and GAP_6.25 (*p* < 0.0001), GAP_0.4 and GAP_3.1 (*p* < 0.0001), GAP_0.4 and GAP_6.25 (*p* < 0.0001), GAP_0.8 and GAP_3.1 (*p* < 0.0001), GAP_0.8 and GAP_6.25 (*p* < 0.0001), GAP_1.6 and GAP_3.1 (*p* = 0.011), GAP_1.6 and GAP_6.25 (*p* < 0.0001), and GAP_3.1 and GAP_6.25 (*p* < 0.0001).

There was no significant correlation between GDT estimated from the CSM values and single gap behavioral GDT (*r* = 0.1566, *p* = 0.4978), between GDT estimated from the CSM values and 80 Hz gap behavioral GDT (*r* = 0.1699, *p* = 0.4617), nor between the behavioral single gap GDT and the behavioral 80 Hz gap GDT (*r* = 0.3994, *p* = 0.0728).

## 4. Discussion

The results of the present study demonstrated that 80 Hz ASSR can be elicited by silent gaps embedded within a broadband noise in people with normal hearing. Significant ASSRs were elicited only by test sound stimuli with silent intervals longer than 1 ms, which was the gap detection threshold derived from behavioral data. The ASSRs elicited by GAP_0.4 and GAP_0.8, which were below the behavioral threshold, were similar to those elicited in the GAP_0 condition, in which a continuous broadband noise was used as a sound stimulus. These results suggest that significant ASSRs were elicited only when the length of the silent gaps exceeded the behavioral threshold. This implies that auditory temporal resolution may be objectively measured using the 80 Hz gap-evoked ASSR.

It is known that stimuli differing in modulation frequencies elicit ASSR originating in different brain areas. Previous studies compared cortical and subcortical neural activity with 40 and 80 Hz amplitude modulated sound stimulations. Herdman et al. (11) measured the ASSR elicited by amplitude modulated tones with three modulation frequencies, 12, 39, and 88 Hz. They estimated the neural sources using multi dipole model, revealing that 88 and 39 Hz amplitude modulated tones mainly elicited subcortical and cortical neural activity, respectively. Additionally, Farahani et al. (18) measured the ASSR elicited by amplitude modulated white noise with the modulation frequencies of 3.91, 19.53, 40.04, and 80.08 Hz and estimated the neural source using a minimum-norm imaging technique. They reached the same conclusions as Herdman et al.—subcortical activity dominant at 80 Hz and cortical activity dominant at 40 Hz. Following these results, it has been widely accepted that the 80 Hz ASSR is elicited mainly in the subcortical regions.

Regarding the neural center for auditory temporal processing enabling auditory gap detection, previous studies have suggested that auditory cortex rather than brainstem plays a key role (19–22). Ison et al. (19) measured gap detection thresholds in rats by acoustic startle reflex using a white noise with silent gaps. KCl injections caused cortical disruptions in rats, inducing prolonged gap detection thresholds, whereas the disruption of auditory brainstem had little effect on gap detection. The results suggested that cortex plays a major role in auditory temporal processing. Syka et al. (21) also demonstrated that neural activity related to auditory temporal processing was delayed after surgical removal of the rat auditory cortex. Similarly, human studies on patients with damage to cerebral hemispheres reported impaired auditory temporal processing regarding the auditory stimuli presented to the ear contralateral to the damaged hemisphere. Jafari et al. (23) performed Gaps-In-Noise test (GIN test, a form of gap detection test) in patients with right hemisphere infarction, patients with left hemisphere infarction and normal subjects. All participants had normal pure tone audiograms; however, the results indicated that auditory temporal processing was impaired when the GIN test was performed on the ear contralateral to the infarction site. Lavasani et al. (24) found that the GDT became longer in patients with temporal epilepsy with normal pure tone audiograms, indicating that auditory temporal processing was impaired in those people. Moreover, research done by Bamiou et al. (25) suggested that insula plays an important role in auditory temporal processing, since patients with insular hemispheric infarction showed prolonged GDT.

In the present study, we used gap sounds that are thought to be processed in the auditory cortex, yet we obtained clear 80 Hz ASSR which are mostly associated with the brainstem. The results obtained could be interpreted as follows. First, while the 80 Hz ASSR is generally believed to originate primarily from the brainstem, the signal appears to be contaminated with the neural activity in the auditory cortex (18, 26). Therefore, the results obtained in the present study might reflect a portion of 80 Hz ASSR derived from the auditory cortex. Alternatively, the present results could imply that the auditory temporal processing involved in the gap detection occurs at least partially in the brainstem. Galambos et al. (10) first reported that the ASSR amplitude became maximal at the modulation rate of 40 Hz. The 40 Hz ASSR was found to become smaller with sleep, sedation, and anesthesia (27). On the other hand, a higher modulation frequency (70–110 Hz) provided a stable ASSR even during sleep or under sedation (13, 28). The ASSRs elicited by 80 Hz amplitude modulated tones are clinically used as an objective audiometry and are often measured during sleep since the signal-to-noise ratio of 80 Hz ASSR improves during sleep (29). In the present study, the participants were awake during the EEG recording; however, the 80 Hz gap-evoked ASSR may become more prominent during sleep. Eighty hertz ASSR recording during sleep would be especially useful for infants and children who could not stay still during the EEG recording.

Interestingly, the detection threshold of 80 Hz gap inserted sound stimuli was significantly shorter than the normal single gap GDT. While the single gap GDT (mean: 2.76 ms) in the present study was similar to those obtained in the previous studies (30, 31), the regular insertion of gaps at 80 Hz appears to shorten the GDT. Bacon et al. (32) measured detection thresholds of sinusoidal amplitude modulation on a broadband noise and found that normal hearing participants could detect up to 1,024 Hz modulation frequency. The results indicated that the participants could detect the envelope fluctuations of about 1 ms. Moreover, Ross and Pantev (33) measured behavioral GDT and the auditory evoked fields elicited by gaps embedded within 500 Hz tones with 40 Hz amplitude modulation. Normally, the gap detection of pure tones with frequencies between 400 and 1,000 Hz was estimated to be between 6 and 8 ms (34); however, Ross and Pantev's (33) results showed that the detection threshold for the gap embedded within the 40 Hz AM tone was 3 ms and the gap duration of 3 ms elicited significant auditory evoked fields. Similar to the above results, the present study also demonstrated that the detection thresholds for gaps embedded within sounds with repetitive envelope fluctuations became shorter than those embedded within continuous sounds with no repetitive fluctuation (35). One might argue that the insertion of periodic gaps added spectral components corresponding to the stimulation rate (80 Hz) and its harmonics, and consequently the participants might have detected the corresponding spectral components. In the present study, we used white noise segments longer than and equal to the half (6.25 ms) of one 80 Hz cycle and thus the 80 Hz gap inserted sound stimuli had spectral components similar to the control white noise as shown in Figure 2. Another possibility is that the participants might have detected the spectral splatter caused by the steep onset and offset sound envelopes of the gap. However, we used white noise as sound stimuli and the onset and offset of white noise did not give a spectral cue since the spectral splatter was masked by the white noise. Therefore, it is less likely that the participants in the present study detected the spectral changes of test sound stimuli instead of temporal ones.

We found no significant relationship between the GDT estimated from the CSM and the behavioral GDT. All the participants in the present study were young adults and had normal hearing. This could explain why there was little variance in the behavioral GDTs among the participants. Moreover, alertness and motivation of the participants appears to have a stronger impact on the behavioral results than the inter-individual difference. It is necessary to conduct similar studies on people with suspected deterioration of auditory temporal resolution, such as auditory neuropathy patients (36, 37) and the elderly (38).

Recent studies focused their attention on people who have normal pure tone audiogram but struggle to listen to speech signals especially in noisy environments (39, 40). This symptom may derive from impaired temporal processing in the auditory system (41). It remains elusive whether speech perception is significantly correlated with the within-channel gap detection threshold (42). According to Tyler et al. (43) and Snell et al. (44) who used noise bursts as stimulus sounds, GDT and speech perception under noise are significantly correlated, while Strouse et al. (45) and Snell et al. (46) found no significant correlation. Although most of those results were obtained in cross-sectional studies, a longitudinal study by Babkoff and Fostick (38) showed a significant relationship between temporal processing and speech perception even after corrections for auditory level and cognitive ability.

Therefore, the gap-evoked ASSR obtained in the present study may reflect the auditory temporal processing and speech comprehension ability. Many ailments thought to be related to impaired auditory temporal processing, such as hidden hearing loss, auditory processing disorder, and other listening difficulties, are currently diagnosed mainly on the basis of the patients' subjective symptoms. The gap-evoked ASSR can be introduced as an objective diagnostic measure for such cases. Moreover, objective measure of auditory temporal processing can be used as a screening test for children with language and speech developmental delays, allowing early detection and early therapeutic intervention for them. Additionally, in cases of elderly patients with communication problems, measurement of gap-evoked ASSR could potentially help discriminate between patients suffering from dementia and those with impaired auditory temporal processing.

## 5. Conclusions

Significant ASSRs and CSMs were elicited only by test stimuli with gap lengths above the behavioral threshold. The test stimuli with gap lengths below the behavioral threshold elicited ASSRs similar to those elicited by the continuous broadband noise. Eighty Hertz gap-evoked ASSR may provide insights into the neural mechanisms of auditory temporal processing and may be applied to objectively and non-invasively measure auditory temporal resolution in humans.

## Data availability statement

The raw data supporting the conclusions of this article will be made available by the authors, without undue reservation.

## Ethics statement

The studies involving human participants were reviewed and approved by Ethics Committee of the International University of Health and Welfare, School of Medicine. The patients/participants provided their written informed consent to participate in this study.

## Author contributions

HO conceived and designed research. SK, TM, and HO performed the experiments. SK and HO analyzed the data. SK, TM, MP, SM, and HO interpreted the results of experiments. SK prepared the figures and drafted the manuscript. All authors edited and revised the manuscript and approved the final version of the manuscript.

## Supplementary material

Supplementary audio 1 An exemplary sound file of GAP_0.

Supplementary audio 2 An exemplary sound file of GAP_0.4.

Supplementary audio 3 An exemplary sound file of GAP_0.8.

Supplementary audio 4 An exemplary sound file of GAP_1.6.

Supplementary audio 5 An exemplary sound file of GAP_3.1.

Supplementary audio 6 An exemplary sound file of GAP_6.3.

## References

[1] Shannon R V Zeng FG Kamath V Wygonski J Ekelid M. Speech recognition with primarily temporal cues. Science. (1995) 270:303–4. 10.1126/SCIENCE.270.5234.303 7569981

[2] Giannela Samelli A Schochat E. The gaps-in-noise test: gap detection thresholds in normal-hearing young adults. Int J Audiol. (2008) 47:238–45. 10.1080/14992020801908244 18465408

[3] Musiek FE Shinn JB Jirsa R Bamiou DE Baran JA Zaida E. GIN (Gaps-In-Noise) test performance in subjects with confirmed central auditory nervous system involvement. Ear Hear. (2005) 26:608–18. 10.1097/01.aud.0000188069.80699.41 16377996

[4] Uther M Jansen DHJ Huotilainen M Ilmoniemi RJ Näätänen R. Mismatch negativity indexes auditory temporal resolution: evidence from event-related potential (ERP) and event-related field (ERF) recordings. Cogn Brain Res. (2003) 17:685–91. 10.1016/S0926-6410(03)00194-0 14561455

[5] Näätänen R Paavilainen P Rinne T Alho K. The mismatch negativity (MMN) in basic research of central auditory processing: a review. Clin Neurophysiol. (2007) 118:2544–90. 10.1016/J.CLINPH.2007.04.026 17931964

[6] Werner LA Folsom RC Mancl LR Syapin CL. Human auditory brainstem response to temporal gaps in noise. J Speech Lang Hear Res. (2001) 44:737–50. 10.1044/1092-4388(2001/058) 11521768

[7] Parkkonen L Fujiki N Mäkelä JP. Sources of auditory brainstem responses revisited: contribution by magnetoencephalography. Hum Brain Mapp. (2009) 30:1772–82. 10.1002/HBM.20788 19378273 PMC6870971

[8] Kadowaki S Morimoto T Okamoto H. Auditory steady state responses elicited by silent gaps embedded within a broadband noise. BMC Neurosci. (2022) 23:27. 10.1186/s12868-022-00712-0 35524192 PMC9074354

[9] Gutschalk A Mase R Roth R Ille N Rupp A Hähnel S. Deconvolution of 40 Hz steady-state fields reveals two overlapping source activities of the human auditory cortex. Clin Neurophysiol. (1999) 110:856–68. 10.1016/S1388-2457(99)00019-X 10400199

[10] Galambos R Makeig S Talmachoff PJ. A 40-Hz auditory potential recorded from the human scalp. Proc Natl Acad Sci USA. (1981) 78:2643–7. 10.1073/pnas.78.4.2643 6941317 PMC319406

[11] Herdman AT Lins O van Roon P Stapells DR Scherg M Picton TW. Intracerebral sources of human auditory steady-state responses. Brain Topogr. (2002) 15:69–86. 10.1023/A:1021470822922 12537303

[12] Picton TW John MS Dimitrijevic A Purcell D. Human auditory steady-state responses: respuestas auditivas de estado estable en humanos. Int J Audiol. (2009) 42:177–219. 10.3109/14992020309101316 12790346

[13] Aoyagi M Watanabe T Ito T Abe Y. Reliability and frequency specificity of auditory steady-state response detected by phase spectral analysis. J Acoust Soc Am. (2007) 122:EL58. 10.1121/1.2761888 17927308

[14] Levitt H. Transformed up-down methods in psychoacoustics. J Acoust Soc Am. (1971) 49:467–77. 10.1121/1.1912375 5541744

[15] Zeng FG Kong YY Michalewski HJ Starr A. Perceptual consequences of disrupted auditory nerve activity. J Neurophysiol. (2005) 93:3050–63. 10.1152/JN.00985.2004/ASSET/IMAGES/LARGE/Z9K0050545510014.JPEG 15615831

[16] Delorme A Makeig S. EEGLAB: an open source toolbox for analysis of single-trial EEG dynamics including independent component analysis. J Neurosci Methods. (2004) 134:9–21. 10.1016/j.jneumeth.2003.10.009 15102499

[17] Aoyagi M. Auditory steady-state response (ASSR). Audiol Jpn. (2006) 49:135–45. 10.4295/AUDIOLOGY.49.135

[18] Farahani ED Wouters J van Wieringen A. Brain mapping of auditory steady-state responses: a broad view of cortical and subcortical sources. Hum Brain Mapp. (2021) 42:780–96. 10.1002/HBM.25262 33166050 PMC7814770

[19] Ison JR O'Connor K Bowen GP Bocirnea A. Temporal resolution of gaps in noise by the rat is lost with functional decortication. Behav Neurosci. (1991) 105:33–40. 10.1037/0735-7044.105.1.33 2025392

[20] Weible AP Moore AK Liu C Deblander L Wu H Kentros C. perceptual gap detection is mediated by gap termination responses in auditory cortex. Curr Biol. (2014) 24:1447–55. 10.1016/J.CUB.2014.05.031 24980499 PMC4131718

[21] Syka J Rybalko N Mazelová J Druga R. Gap detection threshold in the rat before and after auditory cortex ablation. Hear Res. (2002) 172:151–9. 10.1016/S0378-5955(02)00578-6 12361878

[22] Threlkeld SW Penley SC Rosen GD Fitch RH. Detection of silent gaps in white noise following cortical deactivation in rats. Neuroreport. (2008) 19:893–8. 10.1097/WNR.0b013e3283013d7e 18463508 PMC3955703

[23] Jafari Z Esmaili M Delbari A Mehrpour M Mohajerani MH. Auditory temporal processing deficits in chronic stroke: a comparison of brain damage lateralization effect. J Stroke Cerebrovasc Dis. (2016) 25:1403–10. 10.1016/J.JSTROKECEREBROVASDIS.2016.02.030 27021038

[24] Lavasani AN Mohammadkhani G Motamedi M Karimi LJ Jalaei S Shojaei FS. Auditory temporal processing in patients with temporal lobe epilepsy. Epilepsy Behav. (2016) 60:81–5. 10.1016/J.YEBEH.2016.04.017 27179714

[25] Bamiou DE Musiek FE Stow I Stevens J Cipolotti L Brown MM. Auditory temporal processing deficits in patients with insular stroke. Neurology. (2006) 67:614–9. 10.1212/01.WNL.0000230197.40410.DB 16924014

[26] Tsuchimoto R Kanba S Hirano S Oribe N Ueno T Hirano Y. Reduced high and low frequency gamma synchronization in patients with chronic schizophrenia. Schizophr Res. (2011) 133:99–105. 10.1016/J.SCHRES.2011.07.020 21849245

[27] Picton TW Vajsar J Rodriguez R Campbell KB. Reliability estimates for steady-state evoked potentials. Electroencephalogr Clin Neurophysiol. (1987) 68:119–31. 10.1016/0168-5597(87)90039-6 2435528

[28] Rance G Rickards FW Cohen LT De Vidi S Clark GM. The automated prediction of hearing thresholds in sleeping subjects using auditory steady-state evoked potentials. Ear Hear. (1995) 16:499–507. 10.1097/00003446-199510000-00006 8654904

[29] Levi EC Folsom RC Dobie RA. Amplitude-modulation following response (AMFR): effects of modulation rate, carrier frequency, age, and state. Hear Res. (1993) 68:42–52. 10.1016/0378-5955(93)90063-7 8376214

[30] Green DM Forrest TG. Temporal gaps in noise and sinusoids. J Acoust Soc Am. (1998) 86:961. 10.1121/1.398731 2794249

[31] He N-J Horwitz AR Dubno JR Mills JH. Psychometric functions for gap detection in noise measured from young and aged subjects. J Acoust Soc Am. (1999) 106:966. 10.1121/1.427109 10462802

[32] Bacon SP Viemeister NF. Temporal modulation transfer functions in normal-hearing and hearing-impaired listeners. Int J Audiol. (1985) 24:117–34. 10.3109/00206098509081545 3994589

[33] Ross B Pantev C. Auditory steady-state responses reveal amplitude modulation gap detection thresholds. J Acoust Soc Am. (2004) 115:2193. 10.1121/1.1694996 15139631

[34] Moore BCJ Peters RW Glasberg BR. Detection of temporal gaps in sinusoids: effects of frequency and level. J Acoust Soc Am. (1993) 93:1563. 10.1121/1.406815 8473610

[35] Plack CJ. The Sense of Hearing. London: Routledge. (2018). p. 338. 10.4324/9781315208145

[36] Kumar Narne V Alain C Hospital B. Temporal processing and speech perception in noise by listeners with auditory neuropathy. PLoS ONE. (2013) 8:e55995. 10.1371/JOURNAL.PONE.0055995 23409105 PMC3567005

[37] Narne VK Vanaja CS. Perception of speech with envelope enhancement in individuals with auditory neuropathy and simulated loss of temporal modulation processing. Int J Audiol. (2009) 48:700–7. 10.1080/14992020902931574 19626513

[38] Babkoff H Fostick L. Age-related changes in auditory processing and speech perception: cross-sectional and longitudinal analyses. Eur J Ageing. (2017) 14:269–81. 10.1007/S10433-017-0410-Y/METRICS 28936137 PMC5587455

[39] Moore DR Ferguson MA Edmondson-Jones AM Ratib S Riley A. Nature of auditory processing disorder in children. Pediatrics. (2010) 126:e382–90. 10.1542/PEDS.2009-2826 20660546

[40] Sharma M Purdy SC Kelly AS. Comorbidity of auditory processing, language, and reading disorders. J Speech Lang Hear Res. (2009) 52:706–22. 10.1044/1092-4388(2008/07-0226) 19064904

[41] Phillips DP Comeau M Andrus JN. Auditory temporal gap detection in children with and without auditory processing disorder. J Am Acad Audiol. (2010) 21:404–8. 10.3766/jaaa.21.6.5 20701837

[42] Grose JH III JWH Buss E Hatch D. Gap detection for similar and dissimilar gap markers. J Acoust Soc Am. (2001) 109:1587. 10.1121/1.1354983 11325129

[43] Tyler RS Summerfield Q Wood EJ Fernandes MA. Psychoacoustic and phonetic temporal processing in normal and hearing-impaired listenersa). J Acoust Soc Am. (1982) 72:740–52. 10.1121/1.388254 7130532

[44] Snell KB Mapes FM Hickman ED Frisina DR. Word recognition in competing babble and the effects of age, temporal processing, and absolute sensitivity. J Acoust Soc Am. (2002) 112:720–7. 10.1121/1.1487841 12186051

[45] Strouse A Ashmead DH Ohde RN Grantham DW. Temporal processing in the aging auditory system. J Acoust Soc Am. (1998) 104:2385–99. 10.1121/1.423748 10491702

[46] Snell KB Frisina DR. Relationships among age-related differences in gap detection and word recognition. J Acoust Soc Am. (2000) 107:1615–26. 10.1121/1.428446 10738815

